# The BEST Dataset of Language Proficiency

**DOI:** 10.3389/fpsyg.2017.00522

**Published:** 2017-04-06

**Authors:** Angela de Bruin, Manuel Carreiras, Jon Andoni Duñabeitia

**Affiliations:** ^1^Basque Center on Cognition, Brain and LanguageDonostia, Spain; ^2^Departamento de Lengua Vasca y Comunicación, University of the Basque CountryBilbao, Spain; ^3^Ikerbasque, Basque Foundation for ScienceBilbao, Spain

**Keywords:** Basque language, Spanish language, English language, proficiency test, multilingualism, language skills, Basque Country

## Introduction

Researchers investigating processes stemming from or related to multilingualism often face the challenge of correctly characterizing their multilingual samples in terms of language use, proficiency, dominance, and exposure. This is typically done by using a variety of objective (e.g., normed tests) and/or subjective (e.g., self-reports) measures which tend to vary largely across laboratories and studies, since to date the existence of a comprehensive set of measures and norms is limited to certain language combinations (see Gollan et al., 2012). The current study provides a compelling dataset of norms obtained from a large number of Spanish–Basque–English multilinguals from the Basque Country that will facilitate participant selection and classification as a function of their background and language skills.

Contrary to Spanish and English, the Basque language is not a member of the Indo-European language family. It is widely used within the autonomous communities of the Basque Country and Navarra, both located in northern Spain, as well as in the French Pyrénées-Atlantiques. The current study focuses on multilinguals living in the Spanish autonomous community of the Basque Country, an area containing a wealth of bilingual speakers of Spanish and Basque, the two co-official languages, who also know English as a foreign language to a certain extent. Many Basque adults grew up speaking Basque and/or Spanish at home and subsequently received education in one or both of these languages. Additionally, English has been taught as part of the Spanish academic curriculum from the early 1970s, and nearly everyone in the Basque Country below the age of 50 has been exposed to English at school. For this reason, the majority of young and middle-aged Basque adults are better characterized as multilinguals than as bilinguals solely. However, this multilingual population’s knowledge of languages is not homogeneous. While some multilinguals are Spanish dominant, others use Spanish and Basque in a more balanced way or are more dominant in Basque. Furthermore, the exposure to and proficiency in English varies greatly.

The complex linguistic reality of the Basque Country provides researchers with an ideal environment to investigate aspects related to multilingualism. For this reason, recent years have witnessed an exponential increase in the number of psycholinguistic studies on Basque bi-/multilinguals exploring semantic (e.g., Perea et al., 2008), syntactic (e.g., Díaz et al., 2016), lexical (e.g., Duñabeitia et al., 2010b), and ortho-phonological processes (e.g., Casaponsa et al., 2015). Besides, recent studies have also focused on Basque multilinguals in order to explore language-mediated domain-general cognitive processes such as attention (e.g., see Antón et al., 2014, 2016; Duñabeitia et al., 2014) or learning (e.g., Cenoz, 1998). Given this increased presence of studies with Basque multilinguals, some efforts have been made to provide researchers with databases that allow for the creation of adequately characterized research materials (e.g., Perea et al., 2006; Duñabeitia et al., 2010a; Acha et al., 2014).

In order to study Basque multilinguals, and over and above creating adequately controlled materials, one also needs to characterize the different types of participants that constitute the test samples. In the current Data Report we introduce the BEST dataset, which presents data from 650 adult participants from the Basque Country who completed a series of **B**asque, **E**nglish, and **S**panish **t**ests (hence the name BEST) as a proxy for measuring their language skills. The BEST dataset is the result of a collaborative project developed at the Basque Center on Cognition, Brain and Language (BCBL) with the aim of providing researchers from the Basque Country with a series of norms that can be used to better characterize the test samples. Here we present both the range of scores per task, the quantile distribution of these scores, as well as a cluster analysis grouping participants according to the different measurements. This database and the associated materials that include various objective and subjective measures commonly used in psycholinguistic research can be used for future studies aiming to test multilingual samples from the Basque Country.

Self-ratings are an easy and often-used method to assess participants’ proficiency level. Although self-ratings have been found to correlate with more objective proficiency measures (see Marian et al., 2007), they have also been criticized, as participants may over- or underestimate their own proficiency (e.g., MacIntyre et al., 1997). Thus, self-ratings should not be taken as the unique index of language use and proficiency, although they provide useful supplementary information (see Gollan et al., 2012). For this reason, our dataset avoids the subjectivity of only using self-assessed proficiency measurements by combining a range of objective and subjective proficiency measurements. Using multiple tasks to assess proficiency offers a more comprehensive understanding of the participants’ actual language proficiency.

The BEST dataset includes information from four different subtests divided in three language blocks. We report two objective measures that cover different aspects related to vocabulary knowledge, word production and visual word identification. Firstly, following the idea of the Multilingual Naming Test (Gollan et al., 2012), we designed a vocabulary test that was completed by the participants in the three languages. Secondly, participants also completed a series of lexical decision tests (one per language) similar to the LexTALE created by Lemhöfer and Broersma (2012). They had to decide whether each letter string corresponded to an existing word in the target language or not. Thirdly, and following the recommendations of Gollan et al. (2012), all participants underwent a short semi-structured interview guided by a multilingual linguist with experience in assessing language proficiency who provided a score of the participants’ language skills in each language. And lastly, participants completed a short questionnaire about language history and knowledge adapted from other questionnaires previously used in the literature (e.g., Marian et al., 2007). Below we present detailed information about these tests, which can be accessed together with the normative data via https://figshare.com/s/2b377367585a7e5353fb and http://hdl.handle.net/10810/20563.

## Participants

A group of 650 (435 female) participants completed various language proficiency measurements. Their ages ranged from 18 to 50 years (mean = 25.02, *SD* = 5.58). The maximum level of education achieved at the time of testing ranged from high school to university, although the majority of participants (80%) reached a higher level of education (professional training, university, or a postgraduate degree). All participants were Spanish-Basque-English trilinguals and they had acquired Basque and Spanish before the age of six (mean AoA_Spanish_ = 0.67, *SD* = 1.55; mean AoA_Basque_ = 1.68, *SD* = 1.81). On average, English was acquired at a later age (mean AoA_English_ = 6.37, *SD* = 2.49), but all participants reported having acquired English at or before age 12. Regarding the dialectal variations of Basque, the majority of participants reported using either the standard Batua Basque form (54%) or the Gipuzkoan dialect corresponding to the region in which the current study was conducted (38%). An additional six percent of participants reported using a Biscayan or Western dialect while 1% used an upper Navarrese dialect. Although more participants took part in some of the tasks, we only included participants who completed all measurements in the final BEST dataset.

## Tasks and Procedure

Data were collected over a period of 18 months, starting from January 2015 and ending in June 2016. Participants first registered and completed the questionnaire aimed at gathering the subjective measurements and the LexTALE tests using the online platform created for this purpose^1^. After this, they came to the laboratories where they individually completed the picture-naming tests and underwent the semi-structured interview. All participants provided signed consent forms prior to completing the battery of tests, which had been previously validated by the BCBL Ethics Committee. The materials for all tasks can be found in the public repository deposits^2,^^3^.

### Self-rated Proficiency and Exposure

Self-rated proficiency and exposure scores were collected as part of an abridged version of the Language Experience and Proficiency Questionnaire (Marian et al., 2007). Participants were asked to rate their proficiency in Spanish, Basque, and English on a scale from 0 (‘lowest level’) to 10 (‘native or native-like level’) at the general level. Similarly, participants rated their estimated percentage of exposure to each of the three languages on a scale from 0 (‘never’) to 100 (‘always’).

### Interview

Participants completed a short semi-structured oral proficiency interview in each of their three languages (cf., Gollan et al., 2012). This 5-min interview consisted of a set of questions ranging in difficulty and requiring the participant to use different types of grammatical constructions (e.g., questions requiring different tenses). The interview was conducted and assessed by a group of linguists who were native speakers of Basque and Spanish with high proficiency in English. One linguist evaluated each participant, but a total of four linguists with previous professional experience in assessing linguistic competence took part in the process. The scoring was based on a Likert-like scale from 1 (‘lowest level’) to 5 (‘native or native-like level’).

### Picture Naming

Expressive vocabulary was assessed through a picture-naming task akin to the Multilingual Naming Test (cf., Gollan et al., 2012) but specifically adapted for the three examined languages (Spanish, Basque, and English). The test consisted of 65 pictures corresponding to non-cognate words that had to be named in each of the three languages. All pictures showed common entities belonging to different categories such as animals (24 items) or body parts (8 items). Participants took approximately 10 min to complete each language version, and the score per language ranged from 0 to 65. The pictures were taken from the MultiPic database (Duñabeitia et al., 2017). The order of the picture naming tasks was Spanish–Basque–English.

### LexTALE

All participants completed three online versions of LexTALE, a short lexical decision test that has been shown to provide good estimates of language knowledge (cf. Lemhöfer and Broersma, 2012). The order of the LexTALE tasks was Spanish-Basque-English. In the English version of the test, participants were presented with 60 items (40 words, 20 non-words) and they were asked to click on the corresponding button to indicate whether the item was an existing English word or not. For the Spanish version, we used LexTALE-Esp (Izura et al., 2014), which presents participants with 60 real Spanish words and 30 non-words and follows the same rationale and procedure as the original English version. A Basque version of LexTALE was developed for the same purposes following the same validation process described in the original studies. The Basque LexTALE was created in collaboration with three linguists and includes several relatively difficult non-words in order to increase the diagnostic power. While the inclusion of such non-words is restricted to few instances in the English and Spanish versions, we decided to include several exemplars after piloting the Basque version with a larger set of items and using point-biserial correlation analyses to exclude items with low diagnostic power. The final version of the Basque LexTALE comprised 75 items (50 real Basque words and 25 non-words). Thus, the ratio of words versus non-words was kept consistent across the three languages. Some items in the LexTALE tests can furthermore be considered (non-identical) cognates with the other two languages. Test scores for the three versions of the test are based on the percentages of accurate responses to words and non-words, corrected for the unequal number of words and non-words in the test. Hence, the final score in each language resulted from averaging the percentages of correct responses separately obtained for words and non-words, and is provided in terms of percentages.

## Dataset Overview and Description

**FIGURE 1:**
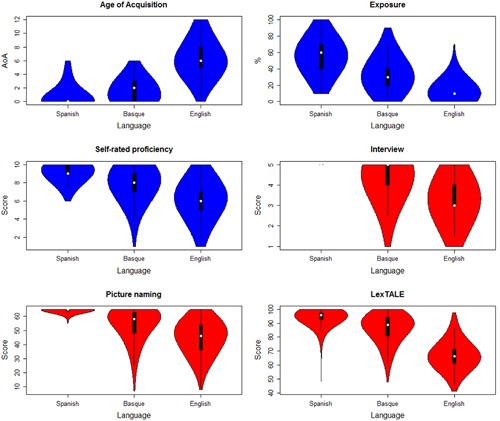
**Violin plots showing the distribution of age of acquisition, language exposure, self-rated proficiency, interview, picture naming, and LexTALE scores, for all three languages.** A description of all tasks can be found in the section ‘tasks and procedure.’ More details about age of acquisition are provided in the paragraph ‘participants.’ Each violin plot shows the distribution of the values (in blue for subjective measurements and in red for objective measurements) as well as the median as a white dot, the interquartile range as a thick black bar, and the 95% confidence interval as a thin black bar. Spanish interview scores are not present in the plot as all participants obtained the maximum score.

The complete BEST dataset with the raw data per participant and task is available at https://figshare.com/s/2b377367585a7e5353fb and http://hdl.handle.net/10810/20563 in a tab-delimited plain text format and in a Microsoft Excel^®^ spreadsheet. The files contain background information about the participants’ age, gender, maximum education level, and handedness. It furthermore provides the individual values of the self-rated percentage of exposure to each language (from 0 to 100), their self-rated general proficiency (from 0 to 10), the scores resulting from the interviews (from 1 to 5), the number of correctly named items in the picture-naming tests (from 0 to 65), and the scores in the three LexTALE tests (from 0 to 100). The summary of these pieces of information is provided in the violin plots presented in **Figure 1**. Besides, **Table 1** presents the information of the cut-off values for the most representative quantiles of the different variables from the 1st to the 99th percentile in steps of 5.

**Table 1:** Cut-off values per quantile in steps of 5 percentiles for the self-rated proficiency, the self-estimated percentage of exposure, the interview mark, the picture-naming test, and the LexTALE test in each of the three languages.

	Percentile																				
	1	5	10	15	20	25	30	35	40	45	50	55	60	65	70	75	80	85	90	95	99
**Spanish**
Self-rated proficiency	7	8	8	8	9	9	9	9	9	9	9	10	10	10	10	10	10	10	10	10	10
Self-estimated exposure	10	20	30	40	40	40	47	50	50	50	60	60	60	60	70	70	70	80	80	90	100
Interview mark	5	5	5	5	5	5	5	5	5	5	5	5	5	5	5	5	5	5	5	5	5
Picture-naming test	60	62	63	64	64	64	65	65	65	65	65	65	65	65	65	65	65	65	65	65	65
Lextale test	77.5	84.5	88	90	92	92.5	93	94	95	95	96	96	97	97	97	97.5	98	98	99	100	100
**Basque**
Self-rated proficiency	3	5	6	7	7	7	7	8	8	8	8	8	9	9	9	9	10	10	10	10	10
Self-estimated exposure	0	10	10	10	20	20	20	20	30	30	30	30	40	40	40	40	50	50	60	70	80
Interview mark	2	2	3	3	4	4	4	4	4	5	5	5	5	5	5	5	5	5	5	5	5
Picture-naming test	18.5	30.5	38	42	45	48	51	53	56	57	58	59	60	61	62	63	64	64	65	65	65
Lextale Test	57	66	70	75	78	81	83	85	86	88	89	90	91	92	93	94	95	96	97	98	100
**English**
Self-rated proficiency	1	3	4	4	5	5	5	6	6	6	6	7	7	7	7	7	8	8	8	9	10
Self-estimated exposure	0	0	0	0	0	10	10	10	10	10	10	10	10	10	10	10	20	20	20	30	40
Interview mark	1	2	2	2	3	3	3	3	3	3	3	3	4	4	4	4	4	4	4	5	5
Picture-naming test	16.5	23	28	31	34	36	39	41	44	45	46	48	49	51	53	54	55	57	59	62	65
Lextale test	47.5	54	56	59	60	61	62.5	62.5	64	65	66	67.5	69	69	70	71	72.5	75	79	85	93

A clustering procedure using all diagnostic indices (i.e., interview, self-perceived proficiency, LexTALE tests and picture-naming tests) was carried out in order to determine the potential subgroups of people considering their English and Basque linguistic skills. As Spanish proficiency was close to or at ceiling for all participants (see **Figure 1**), only English and Basque scores were included in the clustering analysis. K-means was used as a partitioning method for splitting the whole scaled dataset in different clusters. After inspection of the data, and according to the majority rule (namely, the highest number of indices proposing a clustering solution), the best number of clusters was set to 2, indicating that the whole set of participants could be adequately separated in two main subgroups (see Supplementary Figure 1).

Interestingly, the general 2-clusters classification was in agreement with the individual results of parallel clustering procedures carried out on each specific index separately, as shown by the relatively high agreement values obtained (Cohen’s kappa with interview = 0.529; Cohen’s kappa with self-perceived proficiency = 0.440; Cohen’s kappa with LexTALE = 0.479; Cohen’s kappa with picture-naming test = 0.565). However, while there was a relatively high agreement with the individual indices, the results also suggest that the clustering method based on the four measurements was an optimal solution improving any clustering solely based on one individual measurement. In fact, the level of convergence among the clustering solutions individually provided by each index without taking into account the whole set resulted in a mean kappa of 0.382, suggesting only fair agreement (see Landis and Koch, 1977). This result suggests that a combination of measures is preferred over an index obtained from a unitary source, in line with the conclusion of the study by Gollan et al. (2012), who advocated for the use of a multi-measure approach to better estimate multilinguals’ language skills.

Visual inspection of the data points in the clusters and the scores on individual tests suggests that Cluster 1 (in red in Supplementary Figure 1) comprises multilinguals with an overall medium-to-high level of Basque proficiency combined with an English proficiency level ranging from low to high. Cluster 2 (in blue in Supplementary Figure 1) encompasses participants with low-to-medium Basque proficiency, regardless of their English proficiency, which varied from low to high. Thus, the division in clusters is largely based on Basque proficiency with a wide range of English proficiency in both clusters. This is not a surprising finding, given that the age of acquisition of Basque was earlier than the age of acquisition of English, and that Basque is a contextually present language in the Basque Country while English is a foreign language whose presence is mainly restricted to academic contexts.

The usefulness of a multi-dimensional battery is also self-evident when considering the correlations between the objective and subjective proficiency measurements (see Supplementary Figure 2 for correlations between all proficiency measurements as well as correlations between age of acquisition, exposure, and proficiency per language). Regarding AoA, only Basque and Spanish but not English scores correlated significantly with proficiency. In terms of proficiency measurements in Basque, all correlations ranged between *r* = 0.55 (self-rated and LexTALE) and *r* = 0.82 (interview and picture-naming). For English, correlations followed the same pattern, although they were more modest, ranging between *r* = 0.30 (self-rated and LexTALE) and *r* = 0.73 (interview and picture-naming). Again, these results demonstrate that one measure is not enough to capture the idiosyncrasy of the complexity of multilingualism, and they make a plea for a multi-dimensional approach.

## Conclusion

Summarizing, the BEST dataset consists of the individual scores from a large group of multilinguals from the Basque Country who completed language proficiency measurements in Spanish, Basque, and English. While little variety was observed for the different indices of Spanish proficiency, participants showed a wide range of proficiency scores for Basque and English. Our cluster analysis showed that this multilingual group could be divided into two main subgroups mainly based on their Basque proficiency (those with low-to-medium proficiency and those with medium-to-high proficiency). Most importantly, our data highlight the importance of using multiple proficiency measurements rather than a single index from a unique test. We found relatively high agreement between the division of participants in clusters as a function of the clustering based on the four measurements and the division of participants in clusters based on each of the individual tests. In contrast, agreement between the divisions in clusters based on each one of the four individual measurements was much lower. Similarly, the correlations between the different tests in each language showed that despite the underlying common aim, the indices provided are complementary and that the additional information provided by each of them is necessary. In line with previous research, some tests correlated quite highly (e.g., Ferré and Brysbaert, 2016), but it is worth noting that none of the correlations were close to ceiling. Furthermore, correlations between self-ratings and some of the objective tests were relatively low, suggesting that self-ratings alone are not an optimal reflection of proficiency (cf. MacIntyre et al., 1997). Hence, taking multiple objective and subjective indices together provides a more complete understanding of the participants’ language proficiencies.

In conclusion, the BEST dataset offers a partial snapshot of the linguistic reality of the Basque Country and these normative data can be used for a better understanding and characterization of the language knowledge and background of multilingual people from this region with a relatively high level of education. The heterogeneity of the large sample tested allows for a good estimation of the language skills in one or several of the three languages explored, regardless of the number of languages known and their level of proficiency in each of them. Furthermore, our analyses show the importance of combining multiple measurements to obtain a more comprehensive understanding of language proficiency.

## Ethics Statement

This study was carried out in accordance with the recommendations of BCBL Ethics Committee with written informed consent from all subjects. All subjects gave written informed consent in accordance with the Declaration of Helsinki. The protocol was approved by the BCBL Ethics Committee.

## Author Contributions

JA and MC developed the idea together and coordinated the data acquisition. AdB and JA analyzed the data. AdB drafted the manuscript and all authors approved the final version after discussing the intellectual content. All authors agreed to be accountable for all aspects of the work.

## Conflict of Interest Statement

The authors declare that the research was conducted in the absence of any commercial or financial relationships that could be construed as a potential conflict of interest.

## Footnotes

1 http://www.bcbl.eu/participa/

2 https://figshare.com/s/2b377367585a7e5353fb

3 http://hdl.handle.net/10810/20563
