# Supplementary material for: The BEST Dataset of Language Proficiency
Source: Front Psychol. 2017 Apr 6;8:522. doi: 10.3389/fpsyg.2017.00522 (PMC5382213; doi:10.3389/fpsyg.2017.00522)
Supplement: Supplementary file 1 [file Data_Sheet_1.DOCX]

**Supplementary Figure 1**. Data partitioning clustering showing two main clusters of participants according to Basque and English proficiency across four measurements (interview, picture-naming test, LexTALE test, and self-rated proficiency).

**
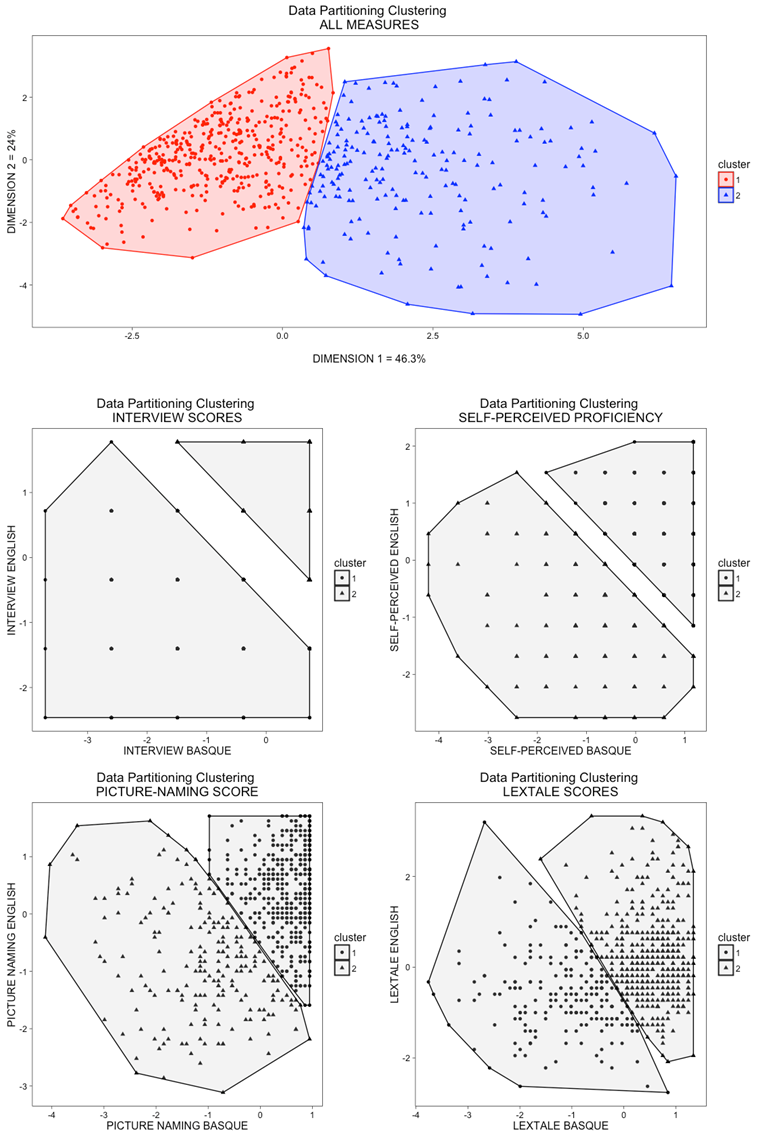
**

**Supplementary Figure 2**. Correlation matrix showing the Pearson’s correlation coefficient (r) between Spanish, Basque, and English self-rated proficiency, interview scores, picture naming scores, and LexTALE scores (matrix 1). Matrices 2, 3, and 4 show the correlations per language between the age of acquisition (AoA), the percentage of exposure to the language, and the different proficiency measurements. Spanish interview scores are not included as all participants scored the maximum score. Values that are crossed out indicate non-significant correlations corrected for multiple comparisons.

**Matrix 1 (all proficiency measurements)**


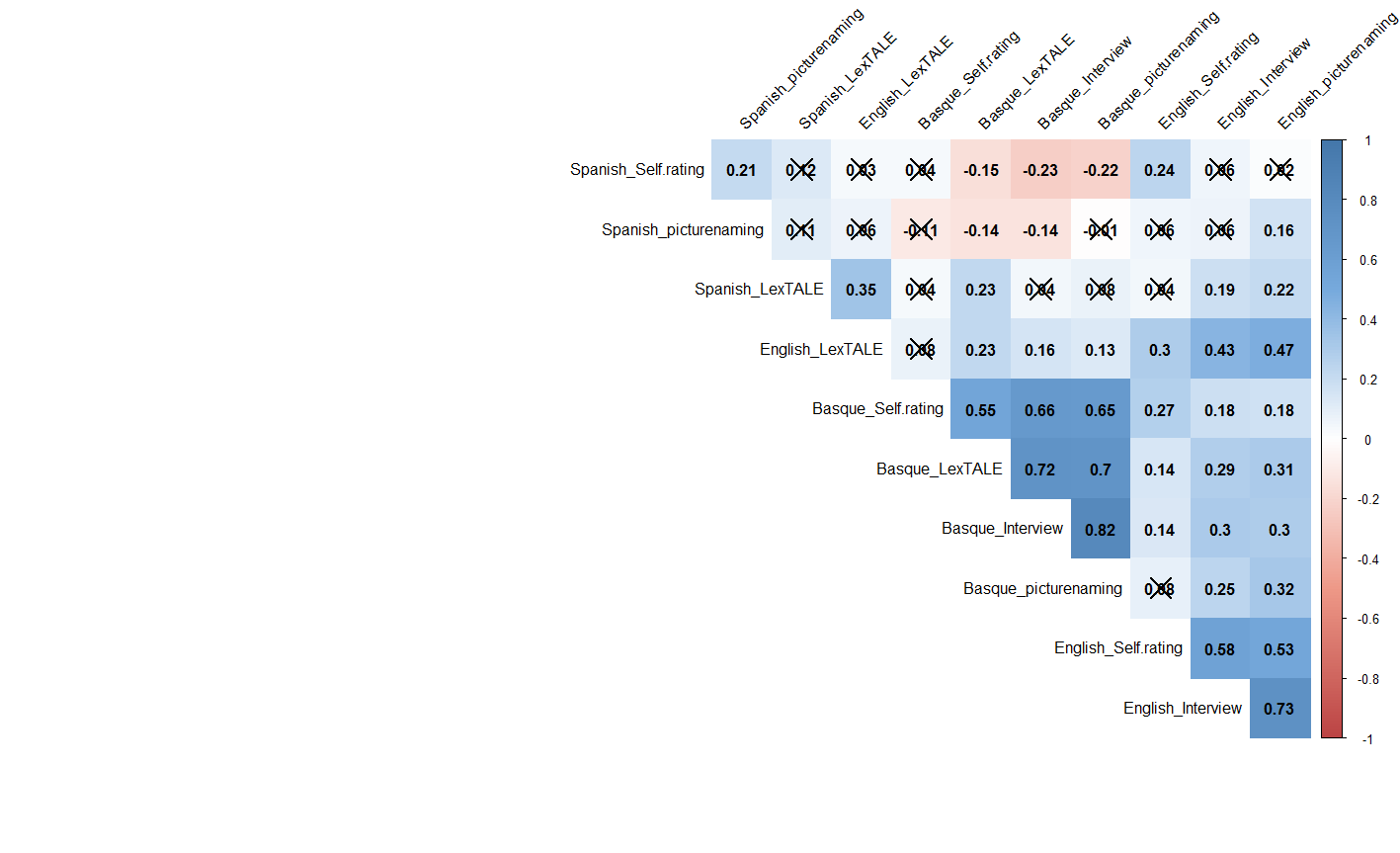


**Matrix 2 (Spanish)**

**
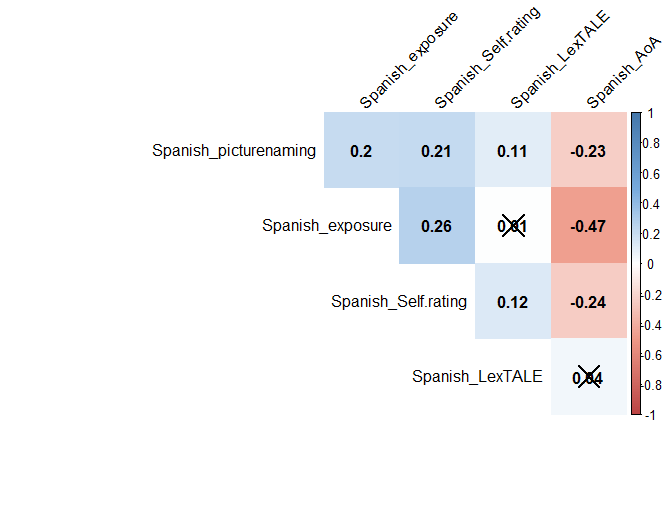
**

**Matrix 3 (Basque)**

**
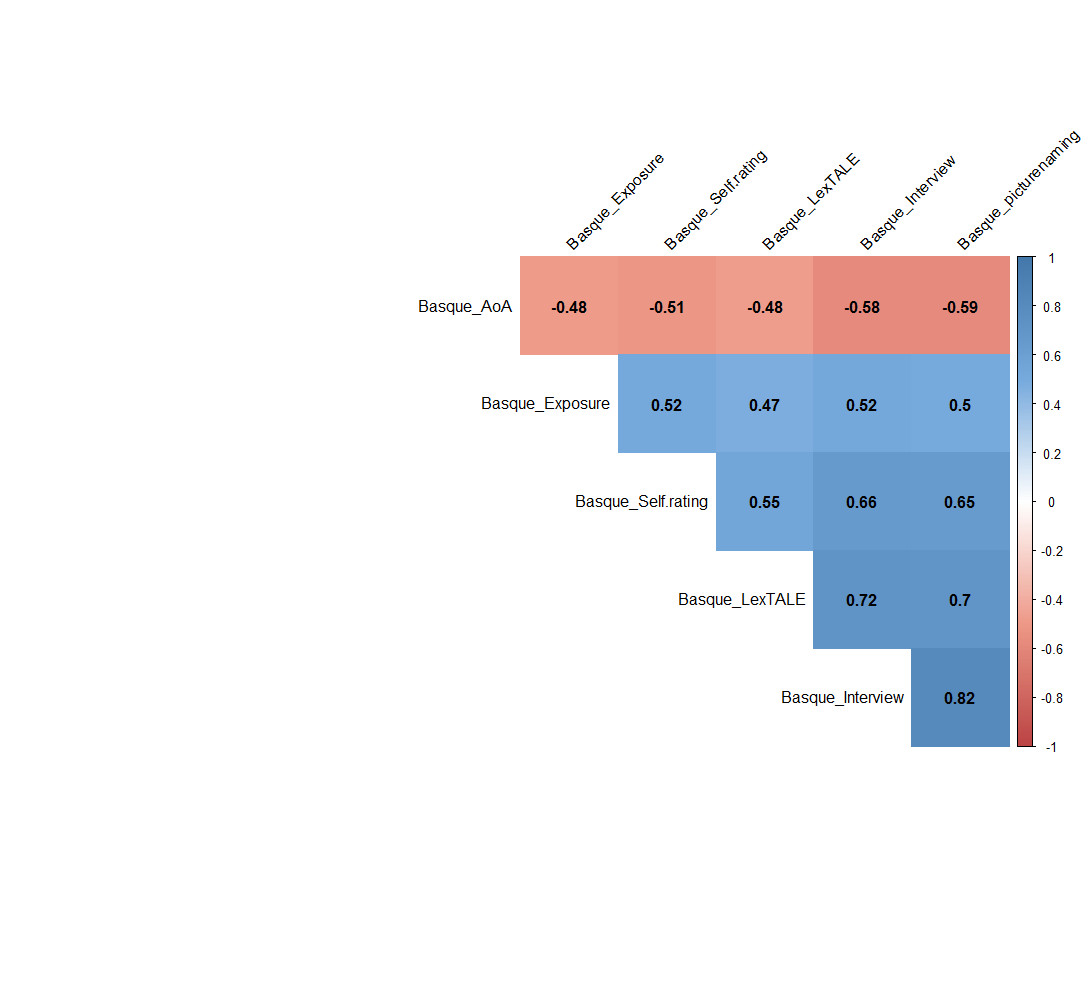
**

**Matrix 4 (English)**

**
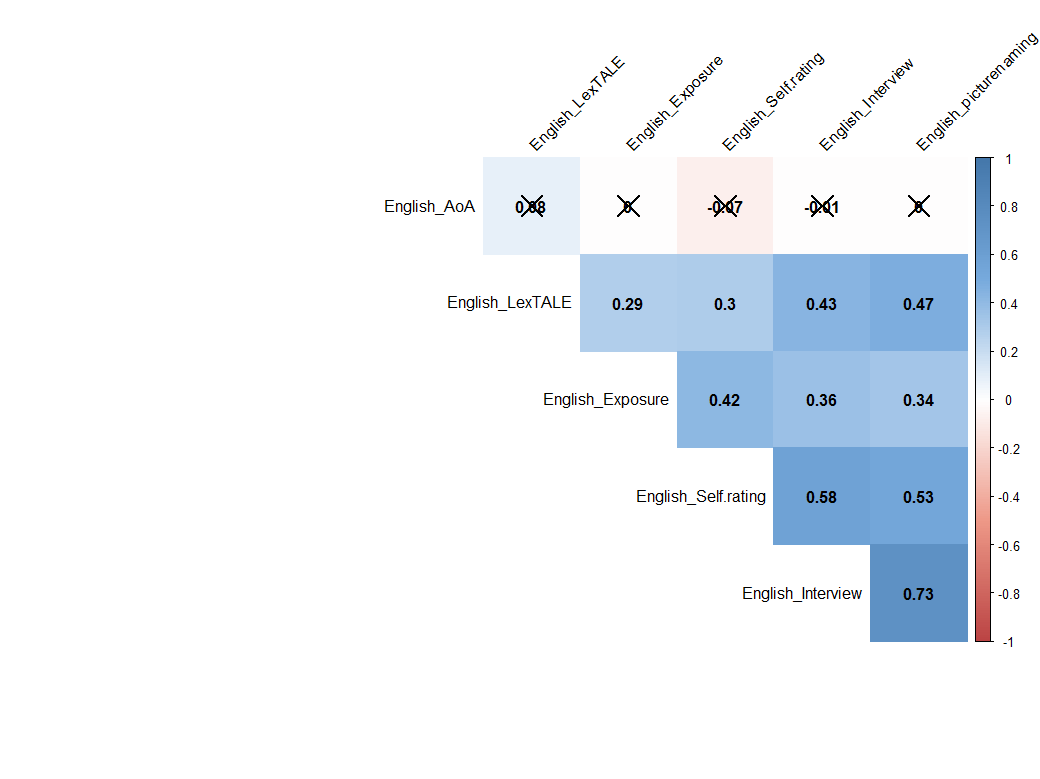
**
